# State of infection prevention and control in Austrian hospitals: data from 81 hospitals completing the WHO Infection Prevention and Control Assessment Framework (IPCAF)

**DOI:** 10.1186/s13756-025-01532-7

**Published:** 2025-03-04

**Authors:** Ferenc Darius Rüther, Andrea Grisold, Agnes Wechsler-Fördös, Alexander Gropmann, Michael Behnke, Sonja Hansen, Christine Geffers, Seven Johannes Sam Aghdassi

**Affiliations:** 1 Institute of Hygiene and Environmental Medicine, Charité – Universitätsmedizin Berlin, Corporate Member of Freie Universität Berlin and Humboldt-Universität Zu Berlin, Berlin, Germany; 2 National Reference Center for Surveillance of Nosocomial Infections, Berlin, Germany; 3 D&R Institute of Hygiene, Microbiology and Environmental Medicine, Medical University, Graz, Austria; 4 Austrian Society of Hygiene, Microbiology and Preventive Medicine, Vienna, Austria

**Keywords:** Infection control, IPCAF, Survey, Surveillance, Infection control structures

## Abstract

**Background:**

The WHO Infection Prevention and Control Assessment Framework (IPCAF) can be used for systematically evaluating infection prevention and control (IPC) practices in healthcare facilities. In 2018, a survey among Austrian hospitals using the IPCAF revealed an overall high level of IPC implementation. Here, we report the results of a second survey in Austrian hospitals with the IPCAF, to once again evaluate the state of IPC implementation and investigate potential developments since 2018.

**Methods:**

A total of 139 Austrian acute care hospitals participating in the German surveillance network “KISS” were invited to complete a translated online version of the IPCAF between October 2023 and January 2024. The IPCAF functions like a questionnaire, where each response is assigned a specific point value, enabling the calculation of an overall IPC score. Based on this score, hospitals were categorized into four distinct IPC levels: inadequate, basic, intermediate, and advanced. The aggregated scores were then calculated and compared with the results from 2018.

**Results:**

Complete datasets from 81 hospitals were received and analyzed. The median overall IPCAF score was 645 (interquartile range: 598–685), with 59 hospitals (72.9%) categorized as advanced, and 21 hospitals (25.9%) as intermediate. One hospital (1.2%) fell into the basic category. Questions pertaining to IPC education and training as well as the application of multimodal IPC strategies showed the lowest scores. Compared to 2018, the current median score of 645 was slightly higher (median score 2018: 620; data from 65 hospitals) and the proportion of hospitals with a full-time IPC professional per 250 beds increased markedly by 37 percentage points. However, the most pronounced decrease (median score − 5) was observed for questions on the WHO core component of IPC education and training.

**Conclusions:**

IPC standards in Austria show an overall increasing trend, especially in terms of IPC staffing. However, areas for improvement remain, and hospitals should make efforts to strengthen IPC education and training programs.

**Supplementary Information:**

The online version contains supplementary material available at 10.1186/s13756-025-01532-7.

## Background

To facilitate the implementation of infection prevention and control (IPC) structures and practices at both national and healthcare facility levels, the World Health Organization (WHO) defined key IPC components essential for establishing strong IPC programs [1, 2]. To assist healthcare facilities in systematically evaluating their IPC programs, the WHO introduced the Infection Prevention and Control Assessment Framework (IPCAF) [3]. Designed primarily as a self-assessment tool, the IPCAF enables healthcare facilities to review their IPC standards. Beyond assessment of individual facilities, it also facilitates larger-scale surveys assessing IPC structures across hospitals within a country. Following its introduction in 2018, we used the IPCAF for surveys in Germany and Austria [4, 5]. The results indicated a generally high level of IPC implementation across the surveyed facilities in both countries. Further studies from multiple countries have provided valuable insights into diverse IPC environments, further validating IPCAF as a key tool for IPC assessment [6–14].

A major advantage of the IPCAF is its capacity for repeated use, enabling healthcare facilities to monitor IPC trends and progress over time, especially in response to observed deficiencies. In the context of national surveys, repeat assessments can reveal significant developments at the country level. In a separate report, we published the results of an IPCAF re-assessment in Germany, and found that overall IPC scores remained stable over time [15]. Now, we seek to report on the repeated application of the IPCAF in Austria to describe the current IPC situation in Austrian hospitals and investigate potential developments.

## Methods

The IPCAF is a structured questionnaire designed for healthcare facilities, encompassing eight sections that align with the core components (CC) of IPC as defined by the WHO [1, 2]. These are: IPC program (CC1), IPC guidelines (CC2), IPC education and training (CC3), healthcare-associated infection (HAI) surveillance (CC4), multimodal strategies for implementing IPC interventions (CC5), monitoring/audit of IPC practices and feedback (CC6), workload, staffing, and bed occupancy (CC7) and built environments, materials, and equipment for IPC (CC8). The questionnaire assigns a score to each response, which is then summed up to calculate a score between 0 and 100 for each of the eight components. The combined scores across all components yield the overall IPCAF score, which can range from 0 to 800 points. Based on their total score and in accordance with the IPCAF instructions [3], healthcare facilities are categorized into an inadequate (0–200), basic (201–400), intermediate (401–600) or advanced (601–800) IPC level.

Recently, for a second time after 2018, Austrian and German acute care hospitals registered in the German surveillance network “Krankenhaus-Infektions-Surveillance-System” (KISS), were invited by the German National Reference Centre for Surveillance of Nosocomial Infections (NRC) to participate in an IPC survey using the IPCAF. KISS serves as the national network for monitoring HAI in Germany. Besides German hospitals, a large number of Austrian hospitals participate as well. It is structured into various modules that focus on different settings or patient populations. For the IPCAF, local KISS contact persons of registered hospitals received a link to an online survey platform (LimeSurvey Community Edition Version 5.2.7). Details regarding the survey organization, as well as a translated version of the IPCAF can be found in the recently published article about the IPCAF results of German hospitals [15]. All responses were submitted online between October 10, 2023, and January 15, 2024, with survey data automatically transmitted to the NRC upon submission. In accordance with participant agreements, the collected data were not referenced with other surveillance metrics, such as infection rates, or the consumption of alcoholic hand rub. Additionally, it was agreed upon that comparisons between 2023 and 2018 were made at the level of aggregated data, and not for individual hospitals. Accordingly, no analyses on particular groups of hospitals (e.g., hospitals participating in both survey) was performed.

Only complete datasets were included in the analysis. Overall IPCAF scores, scores for individual CC, and selected questions of interest were evaluated. Given the extensive nature of the IPCAF, we decided to place a focus on questions of CC with a median score of 75 or less (≤ 75% of 100), as these may reflect IPC aspects that are not yet fully developed or implemented. The cut-off of 75% was selected in alignment with the overall IPCAF scoring system, according to which a score of 600 or less (≤ 75% of the maximum score of 800) no longer qualifies hospitals as being at an advanced IPC level. Moreover, we revisited questions reflecting potential areas for improvement that were highlighted in the 2018 survey. A comprehensive overview of all questions and responses is available in the online supplement (Additional file 1). Data from 2023 were compared with the results from 2018, which had been comprehensively reported in a previous publication [5] and are only displayed in this article, when pertinent for interpreting the 2023 findings. Data analysis and visualizations were performed with Microsoft Excel 2019. ChatGPT (GPT-4) by OpenAI was used to generate and refine selected passages of the manuscript.

## Results

Of 139 invited Austrian acute care hospitals, 92 (66.2%) participated and transmitted their responses to the NRC. A total of 11 (12.0%) received responses had to be excluded from the analysis due to missing data. Ultimately, the IPCAF was fully completed and transmitted by 81 hospitals, resulting in an evaluable response rate of 58.3%.

**Fig. 1:**
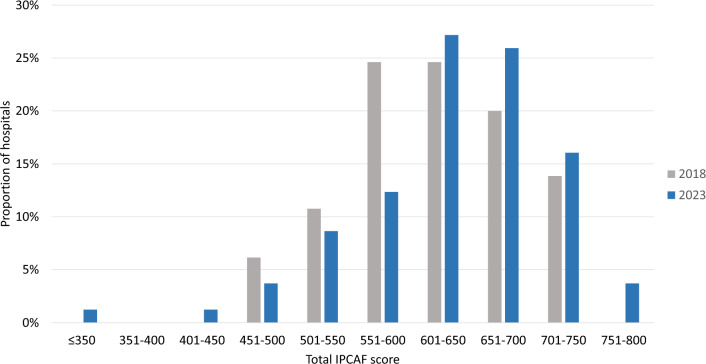
Data from 81 Austrian acute care hospitals in 2023 and 65 hospitals in 2018 that participated in the Infection Prevention and Control Assessment Framework (IPCAF)

When applying the IPC categories, 59 hospitals (72.9%) were classified as advanced, 21 hospitals (25.9%) as intermediate, and one hospital (1.2%) as basic. Notably, no hospital was allocated to an inadequate IPC level. The median overall IPCAF score was 645, with an interquartile range of 598–685, which was slightly higher than in 2018 (median score of 620; data from 65 hospitals). Compared to the results from 2018, there was a slight increase in the proportion of hospitals achieving scores over 600 (Fig. 1).

**Fig. 2:**
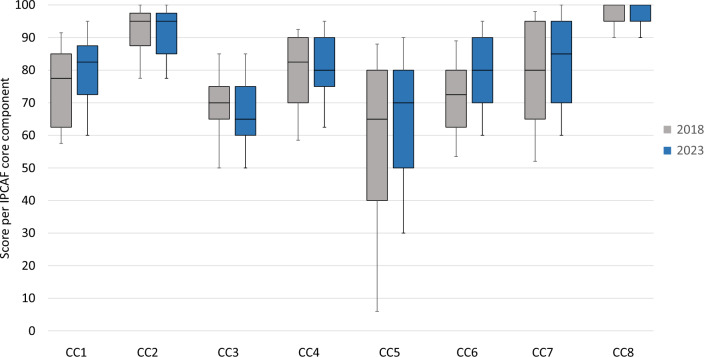
Boxplots displaying the median and range of IPCAF core component scores in 2023 vs. 2018. Data from 81 Austrian acute care hospitals in 2023 and 65 hospitals in 2018. The boxplots display the distribution of scores per core component. The horizontal lines in the box represent the median, the top and bottom of the box represent the interquartile range, the whiskers illustrate the tenth and 90th percentile. Abbreviations: *CC* Core component, *IPCAF* Infection Prevention and Control Assessment Framework

When analyzing the individual CC, the lowest median scores were observed for CC3 (IPC education and training), followed by CC5 (multimodal strategies) (Table 1). In contrast, the highest median scores were recorded for CC2 (IPC guidelines) and CC8 (environment/infrastructure). The range of scores within components (measured between the tenth and 90th percentile) was broadest for CC5 (30–90) and CC7 (workload/staffing) (60–100), and narrowest for CC8 (90–100) and CC2 (77.5–100). There was an overall high level of concordance between CC scores from 2023 and 2018. The highest increase in score (median score + 7.5) was observed in CC6 (monitoring/audit of IPC practices and feedback), while the most pronounced decrease (median score −5) was seen for CC3 (IPC education and training) (Fig. 2).

**Table 1:** Distribution of results of the total IPCAF score and scores per core component

Component	Score					
	Q10	Q25	Q50	Q75	Q90	Mean
CC1	60	72.5	**82.5**	87.5	95	78.3
CC2	77.5	85	**95**	97.5	100	90.6
CC3	50	60	**65**	75	85	67.5
CC4	62.5	75	**80**	90	95	79.0
CC5	30	50	**70**	80	90	64.2
CC6	60	70	**80**	90	95	77.6
CC7	60	70	**85**	95	100	81.0
CC8	90	95	**95**	100	100	96.1
Total	525	597.5	**645**	685	730	634.4

Data from 81 Austrian acute care hospitals in 2023. Abbreviations: *CC* core component (CC1: Infection Prevention and Control (IPC) program, CC2: IPC guidelines, CC3: IPC education and training, CC4: Healthcare-associated infection (HAI) surveillance, CC5: Multimodal strategies for implementation of IPC interventions, CC6: Monitoring/audit of IPC practices and feedback, CC7: Workload, staffing and bed occupancy, CC8: Built environment, materials and equipment for IPC at the facility level); *IPCAF* Infection Prevention and Control Assessment Framework; *Q10* tenth percentile; *Q25* first quartile; *Q50* median (bold numbers); *Q75* third quartile; *Q90* 90th percentile.

For the purpose of a concise presentation and as explained above, a focus will be placed on CC with a median score of 75 or less. Such results were observed for CC3 (IPC education and training) and CC5 (multimodal strategies), with scores of 65 and 70 respectively.

Compared to other CC, the results for CC3 revealed lower levels of implementation across several key areas (Table 2). For instance, only 26% of hospitals reported conducting mandatory IPC training for healthcare workers at least once a year, and only 40% reported offering such training to other personnel. Additionally, not more than 36% of the hospitals included interactive sessions in their training programs. Annual evaluations of training effectiveness were conducted in just 28% of hospitals, and only 20% integrated IPC training into the education of other specialties. Regarding the CC with the second lowest score, CC5, multimodal strategies to implement IPC interventions were used by almost 80% of hospitals, but certain elements like safety climate and culture change, or the formation of a multidisciplinary team were incorporated by fewer hospitals.

**Table 2:** Results from IPCAF core component 3 (IPC education and training) and 5 (multimodal strategies)

		Number (%) IPCAF 2023
CC3, Question 1	**Are there personnel with IPC expertise (in IPC and/or infectious diseases) to lead IPC training?**	
	No	4 (4.9)
	Yes	77 (95.1)
CC3, Question 2	**Are there additional non-IPC personnel with adequate skills to serve as trainers and mentors (for example, link nurses or doctors, champions)?**	
	No	32 (39.5)
	Yes	49 (60.5)
CC3, Question 3	**How frequently do health care workers receive training regarding IPC in your facility?**	
	Never or rarely	1 (1.2)
	New employee orientation only for health care workers	12 (14.8)
	New employee orientation and regular (at least annually) IPC training for health care workers offered but not mandatory	47 (58.0)
	New employee orientation and regular (at least annually) mandatory IPC training for all health care workers	21 (25.9)
CC3, Question 4	**How frequently do cleaners and other personnel directly involved in patient care receive training regarding IPC in your facility?**	
	Never or rarely	3 (3.7)
	New employee orientation only for other personnel	10 (12.3)
	New employee orientation and regular (at least annually) training for other personnel offered but not mandatory	36 (44.4)
	New employee orientation and regular (at least annually) mandatory IPC training for other personnel	32 (39.5)
CC3, Question 5	**Does administrative and managerial staff receive general training regarding IPC in your facility?**	
	No	27 (33.3)
	Yes	54 (66.7)
CC3, Question 6	**How are health care workers and other personnel trained?**	
	No training available	2 (2.5)
	Using written information and/or oral instruction and/or e-learning only	50 (61.7)
	Includes additional interactive training sessions (for example, simulation and/or bedside training)	29 (35.8)
CC3, Question 7	**Are there periodic evaluations of the effectiveness of training programs (for example, hand hygiene audits, other checks on knowledge)?**	
	No	29 (35.8)
	Yes, but not regularly	29 (35.8)
	Yes, regularly (at least annually)	23 (28.4)
CC3, Question 8	**Is IPC training integrated in the clinical practice and training of other specialties (for example, training of surgeons involves aspects of IPC)?**	
	No	30 (37.0)
	Yes, in some disciplines	35 (43.2)
	Yes, in all disciplines	16 (19.8)
CC3, Question 9	**Is there specific IPC training for patients or family members to minimize the potential for health care-associated infections (for example, immunosuppressed patients, patients with invasive devices, patients with multidrug-resistant infections)?**	
	No	52 (64.2)
	Yes	29 (35.8)
CC3, Question 10	**Is ongoing development/education offered for IPC staff (for example, by regularly attending conferences, courses)?**	
	No	0 (0.0)
	Yes	81 (100.0)

CC5, Question 1	**Do you use multimodal strategies to implement IPC interventions?**	
	No	19 (23.5)
	Yes	62 (76.5)
CC5, Question 2	**Do your multimodal strategies include any or all of the following elements:**	
	**- System change**	
	Element not included in multimodal strategies	14 (17.3)
	Interventions to ensure the necessary infrastructure and continuous availability of supplies are in place	33 (40.7)
	Interventions to ensure the necessary infrastructure and continuous availability of supplies are in place and addressing ergonomics and accessibility, such as the best placement of central venous catheter set and tray	34 (42.0)
	**- Education and training**	
	Element not included in multimodal strategies	4 (4.9)
	Written information and/or oral instruction and/or e-learning only	54 (66.7)
	Additional interactive training sessions (includes simulation and/or bedside training)	23 (28.4)
	**- Monitoring and feedback**	
	Element not included in multimodal strategies	18 (22.2)
	Monitoring compliance with process or outcome indicators (for example, audits of hand hygiene or catheter practices)	32 (39.5)
	Monitoring compliance and providing timely feedback of monitoring results to health care workers and key players	31 (38.3)
	**- Communications and reminders**	
	Element not included in multimodal strategies	10 (12.3)
	Reminders, posters, or other advocacy/awareness-raising tools to promote the intervention	58 (71.7)
	Additional methods/initiatives to improve team communication across units and disciplines (for example, by establishing regular case conferences and feedback rounds)	13 (16.0)
	**- Safety climate and culture change**	
	Element not included in multimodal strategies	23 (28.4)
	Managers/leaders show visible support and act as champions and role models, promoting an adaptive approach and strengthening a culture that supports IPC, patient safety and quality	43 (53.1)
	Additionally, teams and individuals are empowered so that they perceive ownership of the intervention (for example, by participatory feedback rounds)	15 (18.5)
CC5, Question 3	**Is a multidisciplinary team used to implement IPC multimodal strategies?**	
	No	31 (38.3)
	Yes	50 (61.7)
CC5, Question 4	**Do you regularly link to colleagues from quality improvement and patient safety to develop and promote IPC multimodal strategies?**	
	No	19 (23.5)
	Yes	62 (76.5)
CC5, Question 5	**Do these strategies include bundles or checklists?**	
	No	17 (21.0)
	Yes	64 (79.0)

Data from 81 Austrian acute care hospitals in 2023. Bold: IPCAF questions of CC3 and CC5

*CC* core component; *IPCAF* Infection Prevention and Control Assessment Framework

When examining potential areas for improvement from the 2018 survey, considerably higher scores in 2023 were observed for questions on the employment of at least one full-time IPC professional per 250 beds (CC1, 63.0% vs. 26.2%) and the use of strategies like bundles and checklists (CC5, 79% vs. 64.6%) (Table 3). Conversely, equal or even lower scores were found for selected questions on IPC training (CC3), surveillance activities (CC4) and staffing ratios (CC7).

**Table 3:** Comparison of results from selected IPCAF questions in 2023 and 2018

		Number (%) IPCAF 2023, n = 81	Number (%) IPCAF 2018, n = 65
CC1, Question 3	**Does the IPC team have at least one full-time IPC professional or equivalent (nurse or doctor working 100% in IPC) available?**		
	No IPC professional available	4 (4.9)	0 (0)
	No, only a part-time IPC professional available	10 (12.3)	16 (24.6)
	Yes, one per > 250 beds	16 (19.8)	32 (49.2)
	Yes, one per ≤ 250 beds	51 (63.0)	17 (26.2)
CC1, Question 6	**Do you have an IPC committee actively supporting the IPC team?**		
	No	12 (14.8)	17 (26.2)
	Yes	69 (85.2)	48 (73.8)
CC3, Question 3	**How frequently do health care workers receive training regarding IPC in your facility?**		
	Never or rarely	1 (1.2)	0 (0)
	New employee orientation only for health care workers	12 (14.8)	6 (9.2)
	New employee orientation and regular (at least annually) IPC training for health care workers offered but not mandatory	47 (58.0)	43 (66.2)
	New employee orientation and regular (at least annually) mandatory IPC training for all health care workers	21 (25.9)	16 (24.6)
CC3, Question 4	**How frequently do cleaners and other personnel directly involved in patient care receive training regarding IPC in your facility?**		
	Never or rarely	3 (3.7)	2 (3.1)
	New employee orientation only for other personnel	10 (12.3)	9 (13.8)
	New employee orientation and regular (at least annually) training for other personnel offered but not mandatory	36 (44.4)	26 (40)
	New employee orientation and regular (at least annually) mandatory IPC training for other personnel	32 (39.5)	28 (43.1)
CC3, Question 6	**How are health care workers and other personnel trained?**		
	No training available	2 (2.5)	0 (0)
	Using written information and/or oral instruction and/or e-learning only	50 (61.7)	40 (61.5)
	Includes additional interactive training sessions (for example, simulation and/or bedside training)	29 (35.8)	25 (38.5)
CC4, Question 6	**In your facility is surveillance conducted for: Colonization or infections caused by multidrug-resistant pathogens according to your local epidemiological situation?**		
	No	20 (24.7)	17 (26.2)
	Yes	61 (75.3)	48 (73.8)
CC4, Question 13	**Do you analyze antimicrobial drug resistance on a regular basis (for example, quarterly/half-yearly/annually)?**		
	No	21 (25.9)	21 (32.3)
	Yes	60 (74.1)	44 (67.7)
CC4, Question 15	**How do you feedback up-to-date surveillance information? (at least annually)**		
	No feedback	4 (4.9)	4 (6.2)
	By written/oral information only	48 (59.3)	42 (64.6)
	By presentation and interactive problem-orientated solution finding	29 (35.8)	19 (29.2)
CC7, Question 2	**Is an agreed (that is, WHO or national) ratio of health care workers to patients maintained across your facility?**		
	No	8 (9.9)	10 (15.4)
	Yes, for staff in less than 50% of units	7 (8.6)	4 (6.2)
	Yes, for staff in more than 50% of units	22 (27.2)	12 (18.5)
	Yes, for all health care workers in the facility	44 (54.3)	39 (60)

Data from 81 Austrian acute care hospitals in 2023. Bold: Selected IPCAF questions

*CC* core component; *IPCAF* Infection Prevention and Control Assessment Framework; *WHO* World Health Organization

## Discussion

This study represents the second large-scale assessment of IPC structures and processes in Austrian hospitals using the IPCAF. With 81 participating hospitals, the survey included a convenience sample of all Austrian acute care hospitals (152 as of 2022, [16]), and therefore provides a robust dataset enabling insights into the current state of IPC practices in Austria. To the best of our knowledge, it is the second national IPC re-assessment utilizing the IPCAF, following the recently reported German survey with 660 participating hospitals [15].

The 58.3% response rate observed in 2023 highlights the overall positive uptake of the IPCAF in Austria. The number of hospitals participating in the IPCAF survey in 2023 was higher than in 2018 (81 vs. 65), indicating a growing interest in the tool. The median overall IPCAF score of 645 corresponds to an advanced level of IPC, and largely coincides with data reported from other high-income countries [4, 7, 10, 12–14]. The median score in 2023 was slightly higher than in 2018 (4% increase). Notably, the proportion of hospitals allocated to an advanced IPC level has increased since 2018, indicating marked progress in IPC structures and practices in Austrian hospitals.

Despite overall improvements, certain CC demonstrated relatively low median scores. For instance, CC3 (IPC education and training) continued to show suboptimal implementation. A potential explanation is the absence of legally mandated IPC training in Austria. This highlights the need for a greater emphasis on IPC education, as numerous studies have underscored its critical role in reducing rates of healthcare-associated infections, particularly when hands-on training for healthcare workers is employed [17–20].

Similar to findings from 2018, scores for CC5 (multimodal strategies for the implementation of IPC interventions) were rather low, which is also consistent with findings from other countries [7, 10, 14]. This may be due to the complexity of the concept and a potential lack of knowledge or experience with the individual modules, indicating the need for more comprehensive education and more clarity around the application of multimodal strategies in IPC.

For CC5 and CC7 (workload, staffing, and bed occupancy), significant inter-hospital variability was observed, illustrating a degree of heterogeneity among Austrian hospitals with regard to these topics. Interestingly, results from the 2023 German IPCAF survey, as well as from the global IPCAF survey in 2019, showed a comparable level of heterogeneity regarding these CC [12, 15]. However, as with any divergent result observed in such survey, heterogeneity in responses may also be attributable to difficulties in interpreting specific questions or ambiguous response options. For example, a positive response to the fifth question of CC5 (“Do these strategies include bundles or checklists”) can mean the use of bundles or the use of checklists or the use of both.

When examining potential areas of improvement that were noted in the 2018 Austrian IPCAF survey, some improvements were observed, though they were limited. For instance, while the respective question in CC5 indicated an increased use of strategies like bundles and checklists (79% vs. 64.6%), the median score of CC5 rose only by three points. Considerable improvements were observed in CC1, including a more than twofold increase in hospitals employing a full-time IPC professional per 250 beds and an increase in the number of hospitals with IPC committees. These advancements might indicate a growing awareness of the importance of IPC and a corresponding willingness to invest in dedicated IPC personnel and structures. This increased commitment reflects a broader recognition of the critical role that robust IPC infrastructure plays in enhancing IPC practices. Conversely, when looking at overall staffing indicators that are addressed in the IPCAF (CC7), no clear trend was observed. This is important to mention, because 20% of hospitals did not maintain the agreed ratio of health care workers to patients in at least 50% of units. Because understaffing is a known risk factor for poorer adherence to IPC measures [21], which cannot be compensated solely by an increase of IPC personnel, and considering the still suboptimal implementation of IPC training, future efforts should focus on better monitoring the workload of health care workers and offering training programs for less qualified personnel. No improvement was observed concerning IPC training-related questions in CC3. For example, the percentage of hospitals providing mandatory annual IPC training for staff has not increased between 2018 and 2023, and the proportion of hospitals employing interactive training methods has declined since 2018. This perceived lack of progress is surprising, given the potential of the COVID-19 pandemic to increase recognition of the importance of comprehensive IPC training. However, it could be related to a more focused training, rather than comprehensively addressing all the required IPC knowledge [22]. Scores of questions on surveillance activities (CC4), such as surveillance of multidrug-resistant organisms and antimicrobial resistance, showed only modest progress, likely due to the substantial time and resources required. This is particularly relevant given that the hospitals participating in the survey were already more integrated into surveillance networks, due to their participation in KISS. Their limited progress in these areas underscores the challenges of enhancing surveillance, even where surveillance programs are already present.

Our study has several limitations. First, data reported in this survey does not stem from a representative sample of Austrian hospitals and consequently, may not fully align with the overall IPC situation in Austrian hospitals. Here, it is particularly relevant that the hospitals invited to this survey, were all participants in the German national surveillance network, potentially reflecting a higher-than-average interest in IPC, which could be even more the case for responding hospitals. However, given the large number of participating hospitals, which are a substantial portion of all Austrian acute care hospitals, cautious national extrapolation of the study findings appears justifiable, although a future survey should consider measures to further increase the representativeness. Second, despite explanatory footnotes, not all participants might have been familiar with some complex concepts that were addressed in the IPCAF, such as multimodal strategies, possibly causing erroneous responses. Third, some questions might have been perceived as sensitive, potentially leading to biased responses despite survey confidentiality. Fourth, datasets from hospitals participating in both surveys were not directly linked, precluding longitudinal analysis. Thus, observed differences in IPCAF scores might reflect cohort variations rather than actual changes. Nonetheless, the high number of participating hospitals in both surveys reduces the risk of such distortions. Lastly, given that the IPCAF is designed for global use, certain questions, such as those related to the built environment (CC8), may be only partially applicable for an IPC assessment in high-income settings, where positive responses are almost universal.

## Conclusion

The repeated application of the IPCAF in Austrian hospitals demonstrated that results of this IPC assessment tool are reproducible. Overall, IPC structures and processes in Austrian hospitals remain at a high level and exhibit stability with a modest trend toward improvement, especially in terms of IPC staffing. However, areas for further improvement remain, particularly in IPC education and training, which is critical for successful IPC programs. Our findings underscore the value of repeated IPC assessments with standardized tools like the IPCAF.

## Supplementary Information

Additional file 1: Results of all single questions from 81 Austrian acute care hospitals that participated in the IPCAF survey 2023

## Data Availability

Not applicable, because all data were collected within the context of the surveillance of healthcare-associated infections conducted in accordance with the German Protection against Infection Act.

## Abbreviations

CC: Core component
HAI: Healthcare-associated infection
IPC: Infection prevention and control
IPCAF: Infection Prevention and Control Assessment Framework
KISS: Krankenhaus-Infektions-Surveillance-System
NRC: National Reference Center for the Surveillance of Nosocomial Infections
WHO: World Health Organization

## Declarations

### Clinical trial number

Not applicable, because all data were collected within the context of the surveillance of healthcare-associated infections conducted in accordance with the German Protection against Infection Act.
