# Supplementary material for: State of infection prevention and control in Austrian hospitals: data from 81 hospitals completing the WHO Infection Prevention and Control Assessment Framework (IPCAF)
Source: Antimicrob Resist Infect Control. 2025 Mar 4;14:18. doi: 10.1186/s13756-025-01532-7 (PMC11877920; doi:10.1186/s13756-025-01532-7)
Supplement: Supplementary file 1 — Additional file 1: Results of all single questions from 81 Austrian acute care hospitals that participated in the IPCAF survey 2023 [file 13756_2025_1532_MOESM1_ESM.pdf]

Results of the Infection Prevention and Control Assessment Framework (IPCAF) in 81 Austrian acute care hospitals

Core component 1: Infection Prevention and Control (IPC) programme

Question/Answer	Number (%)
**1. Do you have an IPC programme?**	
No	5 (6.2)
Yes, without clearly defined objectives	36 (44.4)
Yes, with clearly defined objectives and annual activity plan	40 (49.4)
**2. Is the IPC programme supported by an IPC team comprising of IPC professionals?**	
No	5 (6.2)
Not a team, only an IPC focal person	13 (16.0)
Yes	63 (77.8)
**3. Does the IPC team have at least one full-time IPC professional or equivalent (nurse or doctor working 100% in IPC) available?**	
No IPC professional available	4 (4.9)
No, only a part-time IPC professional available	10 (12.3)
Yes, one per > 250 beds	16 (19.8)
Yes, one per ≤ 250 beds	51 (63.0)
**4. Does the IPC team or focal person have dedicated time for IPC activities?**	
No	15 (18.5)
Yes	66 (81.5)
**5. Does the IPC team include both doctors and nurses?**	
No	3 (3.7)
Yes	78 (96.3)
**6. Do you have an IPC committee actively supporting the IPC team?**	
No	12 (14.8)
Yes	69 (85.2)
**7. Are any of the following professional groups represented/included in the IPC committee?**	
**- Senior facility leadership (for example, administrative director, chief executive officer [CEO], medical director)**	
No	10 (12.3)
Yes	71 (87.7)
**- Senior clinical staff (for example, physician, nurse)**	
No	15 (18.5)
Yes	66 (81.5)
**- Facility management (for example, biosafety, waste, and those tasked with addressing water, sanitation, and hygiene [WASH])**	
No	42 (51.9)
Yes	39 (48.1)
**8. Do you have clearly defined IPC objectives (that is, in specific critical areas)?**	
No	9 (11.1)
Yes, IPC objectives only	20 (24.7)
Yes, IPC objectives and measurable outcome indicators (that is, adequate measures for improvement)	36 (44.4)
Yes, IPC objectives, measurable outcome indicators and set future targets	16 (19.8)

**9. Does the senior facility leadership show clear commitment and support for the IPC programme:**	
**- By an allocated budget specifically for the IPC programme (that is, covering IPC activities, including salaries)?**	
No	26 (32.1)
Yes	55 (67.9)
**- By demonstrable support for IPC objectives and indicators within the facility (for example, at executive level meetings, executive rounds, participation in morbidity and mortality meetings)?**	
No	30 (37.0)
Yes	51 (63.0)
**10. Does your facility have microbiological laboratory support (either present on or off site) for routine day-to-day use?**	
No	1 (1.2)
Yes, but not delivering results reliably (timely and of sufficient quality)	3 (3.7)
Yes, and delivering results reliably (timely and of sufficient quality)	77 (95.1)

Core component 2: Infection Prevention and Control (IPC) guidelines

Question	Number (%)
**1. Does your facility have the expertise (in IPC and/or infectious diseases) for developing or adapting guidelines?**	
No	3 (3.7)
Yes	78 (96.3)
**2. Does your facility have guidelines available for:**	
**- Standard precautions?**	
No	0 (0.0)
Yes	81 (100.0)
**- Hand hygiene?**	
No	0 (0.0)
Yes	81 (100.0)
**- Transmission-based precautions?**	
No	1 (1.2)
Yes	80 (98.8)
**- Outbreak management and preparedness?**	
No	16 (19.8)
Yes	65 (80.2)
**- Prevention of surgical site infection?**	
No	9 (11.1)
Yes	72 (88.9)
**- Prevention of vascular catheter-associated bloodstream infections?**	
No	2 (2.5)
Yes	79 (97.5)
**- Prevention of hospital-acquired pneumonia ([HAP]; all types of HAP, including (but not exclusively) ventilator-associated pneumonia)?**	
No	18 (22.2)
Yes	63 (77.8)
**- Prevention of catheter-associated urinary tract infections?**	
No	2 (2.5)
Yes	79 (97.5)
**- Prevention of transmission of multidrug-resistant (MDR) pathogens?**	
No	0 (0.0)
Yes	81 (100.0)
**- Disinfection and sterilization?**	
No	1 (1.2)

Yes	80 (98.8)
**- Health care worker protection and safety**	
No	3 (3.7)
Yes	78 (96.3)
**- Injection safety?**	
No	3 (3.7)
Yes	78 (96.3)
**- Waste management?**	
No	3 (3.7)
Yes	78 (96.3)
**- Antibiotic stewardship?**	
No	35 (43.2)
Yes	46 (56.8)
**3. Are the guidelines in your facility consistent with national/international guidelines (if they exist)?**	
No	0 (0.0)
Yes	81 (100.0)
**4. Is implementation of the guidelines adapted according to the local needs and resources while maintaining key IPC standards?**	
No	3 (3.7)
Yes	78 (96.3)
**5. Are frontline health care workers involved in both planning and executing the implementation of IPC guidelines in addition to IPC personnel?**	
No	16 (19.8)
Yes	65 (80.2)
**6. Are relevant stakeholders (for example, lead doctors and nurses, hospital managers, quality management) involved in the development and adaptation of the IPC guidelines in addition to IPC personnel?**	
No	17 (21.0)
Yes	64 (79.0)
**7. Do health care workers receive specific training related to new or updated IPC guidelines introduced in the facility?**	
No	16 (19.8)
Yes	65 (80.2)
**8. Do you regularly monitor the implementation of at least some of the IPC guidelines in your facility?**	
No	3 (3.7)
Yes	78 (96.3)

Core component 3: Infection Prevention and Control (IPC) education and training

Question	Number (%)
**1. Are there personnel with the IPC expertise (in IPC and/or infectious diseases) to lead IPC training?**	
No	4 (4.9)
Yes	77 (95.1)
**2. Are there additional non-IPC personnel with adequate skills to serve as trainers and mentors (for example, link nurses or doctors, champions)?**	
No	32 (39.5)
Yes	49 (60.5)

**3. How frequently do health care workers receive training regarding IPC in your facility?**	
Never or rarely	1 (1.2)
New employee orientation only for health care workers	12 (14.8)
New employee orientation and regular (at least annually) IPC training for health care workers offered but not mandatory	47 (58.0)
New employee orientation and regular (at least annually) mandatory IPC training for all health care workers	21 (25.9)
**4. How frequently do cleaners and other personnel directly involved in patient care receive training regarding IPC in your facility?**	
Never or rarely	3 (3.7)
New employee orientation only for other personnel	10 (12.3)
New employee orientation and regular (at least annually) training for other personnel offered but not mandatory	36 (44.4)
New employee orientation and regular (at least annually) mandatory IPC training for other personnel	32 (39.5)
**5. Does administrative and managerial staff receive general training regarding IPC in your facility?**	
No	27 (33.3)
Yes	54 (66.7)
**6. How are health care workers and other personnel trained?**	
No training available	2 (2.5)
Using written information and/or oral instruction and/or e-learning only	50 (61.7)
Includes additional interactive training sessions (for example, simulation and/or bedside training)	29 (35.8)
**7. Are there periodic evaluations of the effectiveness of training programmes (for example, hand hygiene audits, other checks on knowledge)?**	
No	29 (35.8)
Yes, but not regularly	29 (35.8)
Yes, regularly (at least annually)	23 (28.4)
**8. Is IPC training integrated in the clinical practice and training of other specialties (for example, training of surgeons involves aspects of IPC)?**	
No	30 (37.0)
Yes, in some disciplines	35 (43.2)
Yes, in all disciplines	16 (19.8)
**9. Is there specific IPC training for patients or family members to minimize the potential for health care-associated infections (for example, immunosuppressed patients, patients with invasive devices, patients with multidrug-resistant infections)?**	
No	52 (64.2)
Yes	29 (35.8)
**10. Is ongoing development/education offered for IPC staff (for example, by regularly attending conferences, courses)?**	
No	0 (0.0)
Yes	81 (100.0)

Core component 4: Health care-associated infection (HAI) surveillance

Question	Number (%)
**1. Is surveillance a defined component of your IPC programme?**	
No	4 (4.9)

Yes	77 (95.1)
**2. Do you have personnel responsible for surveillance activities?**	
No	5 (6.2)
Yes	76 (93.8)
**3. Have the professionals responsible for surveillance activities been trained in basic epidemiology, surveillance and IPC (that is, capacity to oversee surveillance methods, data management and interpretation)?**	
No	8 (9.9)
Yes	73 (90.1)
**4. Do you have informatics/IT support to conduct your surveillance (for example, equipment, mobile technologies, electronic health records)?**	
No	2 (2.5)
Yes	79 (97.5)
**5. Do you go through a prioritization exercise to determine the HAIs to be targeted for surveillance according to the local context (that is, identifying infections that are major causes of morbidity and mortality in the facility)?**	
No	22 (27.2)
Yes	59 (72.8)
**6. In your facility is surveillance conducted for:**	
**- Surgical site infections?**	
No	4 (4.9)
Yes	77 (95.1)
**- Device-associated infections (for example, catheter-associated urinary tract infections, central line-associated bloodstream infections, peripheral-line associated bloodstream infections, ventilator-associated pneumonia)?**	
No	24 (29.6)
Yes	57 (70.4)
**- Clinically-defined infections (for example, definitions based only on clinical signs or symptoms in the absence of microbiological testing)?**	
No	44 (54.3)
Yes	37 (45.7)
**- Colonization or infections caused by multidrug-resistant pathogens according to your local epidemiological situation?**	
No	20 (24.7)
Yes	61 (75.3)
**- Local priority epidemic-prone infections (for example, norovirus, influenza, tuberculosis [TB], severe acute respiratory syndrome [SARS], Ebola, Lassa fever)?**	
No	24 (29.6)
Yes	57 (70.4)
**- Infections in vulnerable populations (for example, neonates, intensive care unit, immunocompromised, burn patients)?**	
No	25 (30.9)
Yes	56 (69.1)
**- Infections that may affect health care workers in clinical, laboratory, or other settings (for example, hepatitis B or C, human immunodeficiency virus [HIV], influenza)?**	
No	52 (64.2)
Yes	29 (35.8)
**7. Do you regularly evaluate if your surveillance is in line with the current needs and priorities of your facility?**	
No	19 (23.5)
Yes	62 (76.5)

**8. Do you use reliable surveillance case definitions (defined numerator and denominator according to international definitions [e.g. CDC NHSN/ECDC] or if adapted, through an evidence-based adaptation process and expert consultation?**	
No	18 (22.2)
Yes	63 (77.8)
**9. Do you use standardized data collection methods (for example, active prospective surveillance) according to international surveillance protocols (for example, CDC NHSN/ECDC) or if adapted, through an evidence-based adaptation process and expert consultation?**	
No	16 (19.8)
Yes	65 (80.2)
**10. Do you have processes in place to regularly review data quality (for example, assessment of case report forms, review of microbiology results, denominator determination, etc.)?**	
No	21 (25.9)
Yes	60 (74.1)
**11. Do you have adequate microbiology and laboratory capacity to support surveillance?**	
No	1 (1.2)
Yes, can differentiate gram-positive/negative strains but cannot identify pathogens	0 (0.0)
Yes, can reliably identify pathogens (for example, isolate identification) in a timely manner	5 (6.2)
Yes, can reliably identify pathogens and antimicrobial drug resistance patterns (that is, susceptibilities) in a timely manner	75 (92.6)
**12. Are surveillance data used to make tailored unit/facility-based plans for the improvement of IPC practices?**	
No	14 (17.3)
Yes	67 (82.7)
**13. Do you analyze antimicrobial drug resistance on a regular basis (for example, quarterly/half-yearly/annually)?**	
No	21 (25.9)
Yes	60 (74.1)
**14. Do you regularly (for example, quarterly/half-yearly/annually) feedback up-to-date surveillance information to:**	
**- Frontline health care workers (doctors/nurses)?**	
No	13 (16.0)
Yes	68 (84.0)
**- Clinical leaders/heads of department**	
No	6 (7.4)
Yes	75 (92.6)
**- IPC committee**	
No	16 (19.8)
Yes	65 (80.2)
**- Non-clinical management/administration (chief executive officer/chief financial officer)?**	
No	30 (37.0)
Yes	51 (63.0)
**15. How do you feedback up-to-date surveillance information? (at least annually)**	
No feedback	4 (4.9)
By written/oral information only	48 (59.3)
By presentation and interactive problem-orientated solution finding	29 (35.8)

Core component 5: Multimodal strategies for implementation of infection prevention and control (IPC) interventions

Question	Number (%)
**1. Do you use multimodal strategies to implement IPC interventions?**	
No	19 (23.5)
Yes	62 (76.5)
**2. Do your multimodal strategies include any or all of the following elements:**	
**- System change**	
Element not included in multimodal strategies	14 (17.3)
Interventions to ensure the necessary infrastructure and continuous availability of supplies are in place	33 (40.7)
Interventions to ensure the necessary infrastructure and continuous availability of supplies are in place and addressing ergonomics and accessibility, such as the best placement of central venous catheter set and tray	34 (42.0)
**- Education and training**	
Element not included in multimodal strategies	4 (4.9)
Written information and/or oral instruction and/or e-learning only	54 (66.7)
Additional interactive training sessions (includes simulation and/or bedside training)	23 (28.4)
**- Monitoring and feedback**	
Element not included in multimodal strategies	18 (22.2)
Monitoring compliance with process or outcome indicators (for example, audits of hand hygiene or catheter practices)	32 (39.5)
Monitoring compliance and providing timely feedback of monitoring results to health care workers and key players	31 (38.3)
**- Communications and reminders**	
Element not included in multimodal strategies	10 (12.3)
Reminders, posters, or other advocacy/awareness-raising tools to promote the intervention	58 (71.7)
Additional methods/initiatives to improve team communication across units and disciplines (for example, by establishing regular case conferences and feedback rounds)	13 (16.0)
**- Safety climate and culture change**	
Element not included in multimodal strategies	23 (28.4)
Managers/leaders show visible support and act as champions and role models, promoting an adaptive approach and strengthening a culture that supports IPC, patient safety and quality	43 (53.1)
Additionally, teams and individuals are empowered so that they perceive ownership of the intervention (for example, by participatory feedback rounds)	15 (18.5)
**3. Is a multidisciplinary team used to implement IPC multimodal strategies?**	
No	31 (38.3)
Yes	50 (61.7)
**4. Do you regularly link to colleagues from quality improvement and patient safety to develop and promote IPC multimodal strategies?**	
No	19 (23.5)
Yes	62 (76.5)
**5. Do these strategies include bundles or checklists?**	
No	17 (21.0)
Yes	64 (79.0)

Core component 6: Monitoring/audit of IPC practices and feedback

Question	Number (%)
**1. Do you have trained personnel responsible for monitoring/audit of IPC practices and feedback?**	
No	8 (9.9)
Yes	73 (90.1)
**2. Do you have a well-defined monitoring plan with clear goals, targets and activities (including tools to collect data in a systematic way)?**	
No	28 (34.6)
Yes	53 (65.4)
**3. Which processes and indicators do you monitor in your facility?** (Tick all that apply)	
None	0 (0.0)
Hand hygiene compliance (using the WHO hand hygiene observation tool or equivalent)	57 (70.4)
Intravascular catheter insertion and/or care	63 (77.8)
Wound dressing change	54 (66.7)
Transmission-based precautions and isolation to prevent the spread of multidrug resistant organisms (MDRO)	75 (92.6)
Cleaning of the ward environment	73 (90.1)
Disinfection and sterilization of medical equipment/instruments	74 (91.4)
Consumption/usage of alcohol-based handrub or soap	75 (92.6)
Consumption/usage of antimicrobial agents	52 (64.2)
Waste management	62 (76.5)
**4. How frequently is the WHO Hand Hygiene Self-Assessment Framework Survey undertaken?**	
Never	61 (75.3)
Periodically, but no regular schedule	11 (13.6)
At least annually	9 (11.1)
**5. Do you feedback auditing reports (for example, feedback on hand hygiene compliance data or other processes) on the state of the IPC activities/performance?** (Tick all that apply)	
No reporting	0 (0.0)
Yes, within the IPC team	80 (98.8)
Yes, to department leaders and managers in the areas being audited	80 (98.8)
Yes, to frontline health care workers	74 (91.4)
Yes, to the IPC committee or quality of care committees or equivalent	61 (75.3)
Yes, to hospital management and senior administration	68 (84.0)
**6. Is the reporting of monitoring data undertaken regularly (at least annually)?**	
No	3 (3.7)
Yes	78 (96.3)
**7. Are monitoring and feedback of IPC processes and indicators performed in a "blame-free" institutional culture aimed at improvement and behavioural change?**	
No	4 (4.9)
Yes	77 (95.1)
**8. Do you assess safety cultural factors in your facility (for example, by using other surveys such as HSOPSC, SAQ, PSCHO, HSC)**	
No	64 (79.0)
Yes	17 (21.0)

Core component 7: Workload, staffing and bed occupancy

Question	Number (%)
**1. Are appropriate staffing levels assessed in your facility according to patient workload using national standards or a standard staffing needs assessment tool such as the WHO Workload indicators of staffing need method?**	
No	18 (22.2)
Yes	63 (77.8)
**2. Is an agreed (that is, WHO or national) ratio of health care workers to patients maintained across your facility?**	
No	8 (9.9)
Yes, for staff in less than 50% of units	7 (8.6)
Yes, for staff in more than 50% of units	22 (27.2)
Yes, for all health care workers in the facility	44 (54.3)
**3. Is a system in place in your facility to act on the results of the staffing needs assessments when staffing levels are deemed to be too low?**	
No	27 (33.3)
Yes	54 (66.7)
**4. Is the design of wards in your facility in accordance with international standards regarding bed capacity?**	
No	5 (6.2)
Yes, but only in certain departments	22 (27.2)
Yes, for all departments (including emergency department and pediatrics)	54 (66.6)
**5. Is bed occupancy in your facility kept to one patient per bed?**	
No	3 (3.7)
Yes, but only in certain departments	7 (8.6)
Yes, for all units (including emergency departments and pediatrics)	71 (87.7)
**6. Are patients in your facility placed in beds standing in the corridor outside of the room (including beds in the emergency department)?**	
Yes, more frequently than twice a week	3 (3.7)
Yes, less frequently than twice a week	9 (11.1)
No	69 (85.2)
**7. Is adequate spacing of > 1 meter between patient beds ensured in your facility?**	
No	0 (0.0)
Yes, but only in certain departments	11 (13.6)
Yes, for all departments (including emergency department and pediatrics)	70 (86.4)
**8. Is a system in place in your facility to assess and respond when adequate bed capacity is exceeded?**	
No	4 (4.9)
Yes, this is the responsibility of the head of department	36 (44.4)
Yes, this is the responsibility of the hospital administration/management	41 (50.6)

Core component 8: Built environment, materials and equipment for IPC at the facility level

Question	Number (%)
**1. Are water services available at all times and of sufficient quantity for all uses (for example, hand washing, drinking, personal hygiene, medical activities, sterilization, decontamination, cleaning and laundry)?**	
No, available on average < 5 days per week	0 (0.0)
Yes, available on average ≥ 5 days per week or every day but not of sufficient quantity	0 (0.0)

Yes, every day and of sufficient quantity	81 (100.0)
**2. Is a reliable safe drinking water station present and accessible for staff, patients and families at all times and in all locations/wards?**	
No, not available	0 (0.0)
Sometimes, or only in some places or not available for all users	0 (0.0)
Yes, accessible at all times and for all wards/groups	81 (100.0)
**3. Are functioning hand hygiene stations (that is, alcohol-based handrub solution or soap and water and clean single-use towels) available at all points of care?**	
No, not present	0 (0.0)
Yes, stations present, but supplies are not reliably available	0 (0.0)
Yes, with reliably available supplies	81 (100.0)
**4. In your facility, are ≥ 4 toilets or improved latrines available for outpatient settings or ≥ 1 per 20 users for inpatient settings?**	
Less than required number of toilets or latrines available and functioning	2 (2.5)
Sufficient number present but not all functioning	0 (0.0)
Sufficient number present and functioning	79 (97.5)
**5. In your health care facility, is sufficient energy/power supply available at day and night for all uses (for example, pumping and boiling water, sterilization and decontamination, incineration or alternative treatment technologies, electronic medical devices, general lighting of areas where health care procedures are performed to ensure safe provision of health care and lighting of toilet facilities and showers)?**	
No	0 (0.0)
Yes, sometimes or only in some of the mentioned areas	0 (0.0)
Yes, always and in all mentioned areas	81 (100.0)
**6. Is functioning environmental ventilation (natural or mechanical) available in patient care areas?**	
No	1 (1.2)
Yes	80 (98.8)
**7. For floors and horizontal work surfaces, is there an accessible record of cleaning, signed by the cleaners each day?**	
No record of floors and surfaces being cleaned	24 (29.6)
Record exists, but is not completed and signed daily or is outdated	11 (13.6)
Yes, record completed and signed daily	46 (56.8)
**8. Are appropriate and well-maintained materials for cleaning (for example, detergent, mops, buckets, etc.) available?**	
No materials available	0 (0.0)
Yes, available but not well maintained	1 (1.2)
Yes, available and well-maintained	80 (98.8)
**9. Do you have single patient rooms or rooms for cohorting patients with similar pathogens if the number of isolation rooms is insufficient (for example, TB, measles, cholera, Ebola, SARS)?**	
No	4 (4.9)
No single rooms but rather rooms suitable for patient cohorting available	11 (13.6)
Yes, single rooms are available	66 (81.5)
**10. Is PPE available at all times and in sufficient quantity for all uses for all health care workers?**	
No	0 (0.0)
Yes, but not continuously available in sufficient quantities	0 (0.0)
Yes, continuously available in sufficient quantities	81 (100.0)

**11. Do you have functional waste collection containers for non-infectious (general) waste, infectious waste and, sharps waste in close proximity to all waste generation points?**	
No bins or separate sharps disposal	0 (0.0)
Separate bins present but lids missing or more than 3/4 full; only two bins (instead of three); or bins at some but not all waste generation points	2 (2.5)
Yes	79 (97.5)
**12. Is a functional burial pit/fenced waste dump or municipal pick-up available for disposal of non-infectious (non-hazardous/general waste)?**	
No pit or other disposal method used	3 (3.7)
Pit in facility but insufficient dimensions; pits/dumps overfilled or not fenced/locked; or irregular municipal waste pick up	0 (0.0)
Yes	78 (96.3)
**13. Is an incinerator or alternative treatment technology for the treatment of infectious and sharp waste (for example, an autoclave) present (either present on or off site and operated by a licensed waste management service), functional and of a sufficient capacity?**	
No, none present	3 (3.7)
Present, but not functional	0 (0.0)
Yes	78 (96.3)
**14. Is a wastewater treatment system (for example, septic tank followed by drainage pit) present (either on or off site) and functioning reliably?**	
No, not present	3 (3.7)
Yes, but not functioning reliably	0 (0.0)
Yes and functioning reliably	78 (96.3)
**15. Does your health care facility provide a dedicated decontamination area and/or sterile supply department (either present on or off site and operated by a licensed decontamination management service) for the decontamination and sterilization of medical devices and other items/equipment?**	
No, not present	2 (2.5)
Yes, but not functioning reliably	0 (0.0)
Yes and functioning reliably	79 (97.5)
**16. Do you reliably have sterile and disinfected equipment ready for use?**	
No, available on average < five days per week	0 (0.0)
Yes, available on average ≥ five days per week or every day, but not of sufficient quantity	0 (0.0)
Yes, available every day and of sufficient quantity	81 (100.0)
**17. Are disposable items available when necessary? (for example, injection safety devices, examination gloves)**	
No, not available	0 (0.0)
Yes, but only sometimes available	0 (0.0
Yes, continuously available	81 (100.0)
